# Phylogeography Study of *Ammodytes personatus* in Northwestern Pacific: Pleistocene Isolation, Temperature and Current Conducted Secondary Contact

**DOI:** 10.1371/journal.pone.0037425

**Published:** 2012-05-25

**Authors:** Zhiqiang Han, Takashi Yanagimoto, Yaping Zhang, Tianxiang Gao

**Affiliations:** 1 Fishery College, Zhejiang Ocean University, Zhoushan, Zhejiang, China; 2 National Research Institute of Far Seas Fisheries, Yokohama, Japan; 3 State Key Laboratory of Genetic Resources and Evolution, Kunming Institute of Zoology, Chinese Academy of Sciences, Kunming, China; 4 Fishery College, Ocean University of China, Qingdao, Shandong, China; American Museum of Natural History, United States of America

## Abstract

To assess the role of historical process and contemporary factors in shaping population structures in Northwestern Pacific, mitochondrial control region sequences were analyzed to characterize the phylogeography and population structure of the Japanese sand lance *Ammodytes personatus*. A total of 429 individuals sampled from 17 populations through the species' range are sequenced. Two distinct lineages are detected, which might have been divergent in the Sea of Japan and Pacific costal waters of Japanese Island, during the low sea level. Significant genetic structure is revealed between the Kuroshio and Oyashio Currents. However, significant genetic structure is also detected in the Sea of Japan, contracting expected homogenization hypothesis in Tsushima Current. The haplotype frequency of lineages in both sides of Japanese Island and significant genetic structure between north and south groups revealed that the distribution of lineage B and north group were highly limited by the annual sea temperature. The lack of lineage B in Qingdao population with low sea temperature reflects the sea temperature barrier. Lack of genetic structure in the south group and north group populations indicated that ocean currents within groups facilitated the dispersal of *A. personatus*.

## Introduction

Geographical patterns of genetic variation reflect both historical process and present gene flow attributable to the biological characteristics of the organism under study [1]–[2]. A number of mechanisms have been suggested to explain how population structure can evolve in a marine environment without any obvious physical boundary to gene flow (reviewed in [3]). First of all, historical process associated with climatic oscillations through geological time is one of the most important factors in determining the current distribution of species. Climatic changes have had a major influence on the formation of species, the establishment of major intraspecific phylogenetic lineages, and the patterning of the present-day distribution of plant and animal species [4]. Besides historical processes, a number of contemporary-acting evolutionary mechanisms have been recognized as important factors in generating genetic structuring within marine species. Dispersal ability of larva and adult, local adaptation, oceanographic currents, sea temperature, salinity habitat discontinuities and isolation by distances, have been responsible for the population differentiation in marine species [3], [5]–[7].

Previous studies have demonstrated that the genetic variation of marine species in Northwestern Pacific have been heavily influenced by Pleistocene glaciations. Comparative phylogeography studies have revealed the Sea of Japan as glacial refugium for marine species in northwestern Pacific [8]–[10]. The Sea of Japan is a semi-enclosed marginal sea. It is connected southward to the East China Sea through Tsushima Strait, to the Pacific Ocean through Tsugaru Strait, and northward to the Sea of Okhotsk through Soya and Mamiya Straits, all of which are narrow and shallower than 130 m. Many recent works have estimated sea-level changes in the Sea of Japan during the whole Quaternary. During Pleistocene glaciations, the maximum sea level was within the range from –130 m to –140 m. Due to the low sea level, the Sea of Japan was almost isolated from the Pacific Ocean during glaciation events [11]. The communication between the Sea of Japan and Pacific Ocean was interrupted by the low sea level. Thus, the Sea of Japan is thought a good natural setting to investigate some of the effects of sea level change on marine taxa.

Except historical process, in marine environments the geographic structure of populations may be also influenced by contemporary factors. One way to obtain further insight into the factors shaping the genetic structure of populations is to combine genetic data with information on landscape characteristics, referred to as landscape genetics [12]. Landscape genetics approaches have previously yielded new insights into dispersal patterns and population contingency in terrestrial organisms. (reviewed in [13]). In marine environments, landscapes are characterized by quantitative factors such as, temperature and salinity, and qualitative factors, represented by gyres and other oceanographic attributes. Jøgensen et al. have used the landscape genetics approaches to successfully reveal the population genetic of spring – spawning herring in the Baltic Sea [13].

Surface sea temperature in coastal waters of Japanese Island is heavily influenced by the Kuroshio Current and Oyashio Current in the Pacific Ocean side and the Tsushima Current in the Sea of Japan side. In the northwest Pacific Ocean, two western boundary currents, the subtropical warm and saline Kuroshio Current and the subarctic cold and less saline Oyashio Current, meet each other off the east coast of the Japanese Islands. The Kuroshio Current flows along the south coast of the Japanese Islands and toward the east off central Japan as a part of the wind-driven subtropical gyre circulation cell. This current transports tremendous amounts of heat from the low latitudes to the high latitudes, similar to the Gulf Stream. The Oyashio Current is the western component of the Kamchatka–Alaskan Current, and carries cold and high-nutrient surface water to Northwestern Pacific. These two currents produce the mixed water mass region between the Kuroshio and Oyashio Fronts, where the most pronounced latitudinal gradients of these surface temperature (SST) and sea surface salinity (SSS) are observed in the North Pacific [14]. These sharp temperature changes, salinity gradients off the center Japan could limit the dispersal of marine larva. This is supported by the distribution patterns of larval myctophid fishes in the transition zone between the Kuroshio and Oyashio Fronts [15]. According to the limitation of dispersal between currents, a significant portion of the observed genetic variation in transition zone will be expected. However, there is only one warm current that flows along the Sea of Japan coast off Japanese Island. It is the Tsushima Current, a branch of the warm Kuroshio Current. The Tsushima Current supplies a large quantity of heat and transports marine organisms (reviewed in [11]). The connectivity should be high among populations within the Sea of Japan coast off Japanese Island because of this current. Thus, the coastal waters around Japan offer a heterogeneous landscape where the genetic structure of populations could be affected by both ocean currents and sea temperature features. So the Sea of Japan and the Pacific Ocean around Japan appears to provide one of the best settings to study the impact of climatic oscillations and landscape factors on genetic differentiation in marine species.

The Japanese sand lance *Ammodytes personatus* is a common and commercially important species in Northwestern Pacific. Populations of *A. personatus* are distributed along the both sides of coast around Japan and the Yellow Sea. It is a cold-water species and abundant in shallow near shore areas ranging in depth to 100 meters. It rises to the sea surface by night, and buries itself in sand when water temperature increases to 17–20°C during summer in southern and central Japan populations [16]. However, no aestivation is observed in Cape Soya [17]. Aestivation enables *A. personatus*, a cold-water fish, to live at the southern “environmental edge” for this species [18]. During winter, mature adults spawn demersal and adhesive eggs, which adhere to a grain of sands or a pebble in the seabed. The main spawning grounds were located in bays with a high degree of opening; offshore waters were considered to be inadequate for larval survival [19]. Depend on different water temperature, eggs hatch in 13–33 days. The length of the pelagic larval stage is about 30 days [20]. Larvae of *A. personatus* are able to survive for long periods without food after hatching. When being kept without food, time to 50 percent mortality was about 11, 16, and 21 days after hatching at 15.5, 10.5, and 6.5°C, respectively. Horizontal distribution, and possible abundance of larvae, is strongly influenced by tidal currents, oceanography, and wind conditions [21]. These early life-history characteristics indicate that potential larval dispersal of *A. personatus* is high. If larvae could travel on the currents, the connectivity should be high among populations within the current area. No long migration but a micro geographic migration was observed in adults of *A. personatus* [22]. *A. personatus* exhibits considerable genetic, morphological and ecological variation among populations. Okamoto *et al* performed the genetic study on *A. personatus* using horizontal starch gel electrophoresis to analyze genetic structure of species along the Pacific coast of Japan [23]. Clear genetic differences were found between populations north of Iwate Prefecture and those south of Miyagi Prefecture. This genetic geographic distribution is consistent with that of southern and northern groups previously described by morphological studies [24]. The boundary of two groups was consistent with the transition zone between two currents. This might indicate that the cold and warm currents influence the dispersal of *A. personatus*. *A. personatus* around the coastal waters of Japan would therefore seem a well-suited system for testing hypothesis about correlation between genetic differentiation and local environmental variable. In the present study, we assess and compare the phylogeographical pattern of *A. personatus* in different ocean current system by mitochondrial DNA (mtDNA) control region sequences and evaluate the role of historical and present environment factors in shaping the genetic structures of marine species.

## Results

A 532-bp segment of the 5′end of the control region (including a 27 bp segment of the tRNA^pro^) was sequenced in 429 specimens from 17 populations revealing 402 haplotypes. All haplotype sequences were deposited in GenBank, with accession nos JQ913643-JQ914044. Sequence comparisons revealed 140 polymorphic sites (96 parsimony informative) with 105 transitions, 55 transversions and 26 indels. Fifteen haplotypes were shared among populations and three were found in more than one individual, but only in one population. The number of pairwise differences between haplotypes ranged from 1 to 36. The gamma distribution shape parameter was 0.677, indicating moderate mutation rate heterogeneity among sites in *A. personatus*.

**Figure 1:**
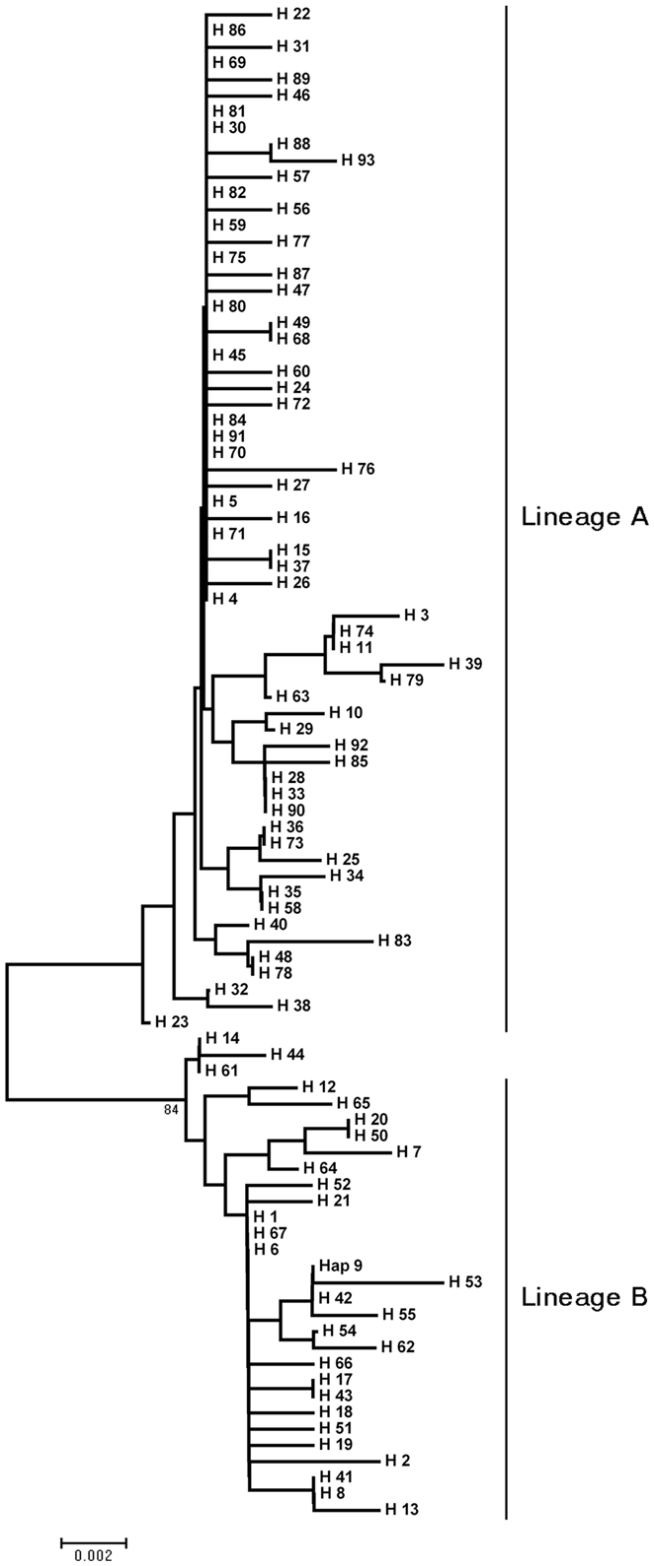
Neighbor-joining tree of transversion haplotypes constructed using Tamura and Nei distances.

**Figure 2:**
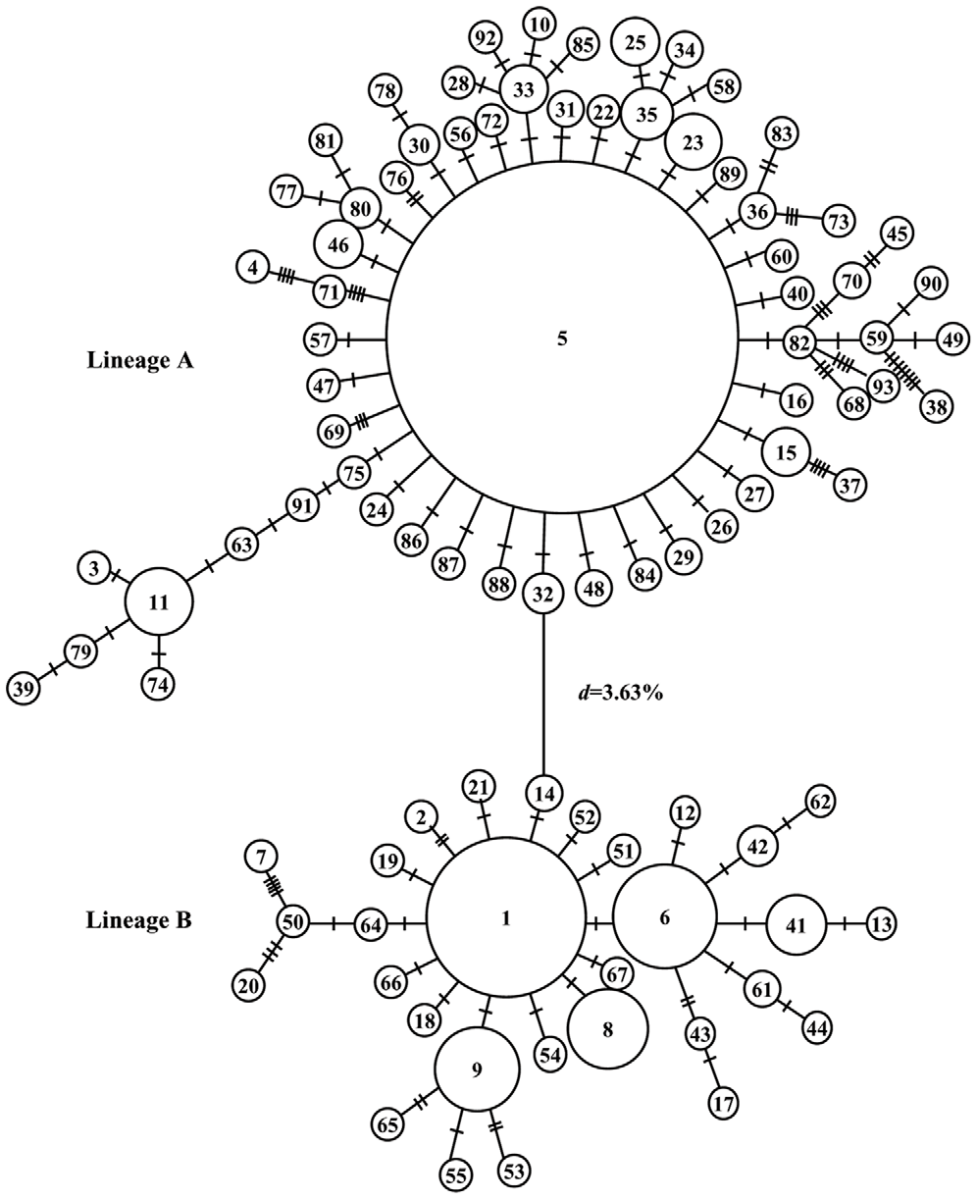
Reduced median-networks showing genetic relationship among control region transversion haplotypes in lineage A and B.

Phylogenetic analysis of haplotypes revealed two distinct lineages (Lineage A and B) in either analysis of NJ tree and haplotype network (Fig. 1; Fig. 2). Net average genetic distance between lineages was 3.63%. Applying sequence divergence rate in control region, the divergence of lineage A and B occurred about 453,000 years ago. Lineage A contained 299 individuals, with haplotype diversity of 0.999±0.001. This lineage had 118 polymorphic sites and nucleotide diversity was 0.0138±0.0072. Lineage B included 130 individuals, with haplotype diversity of 0.999±0.001 and nucleotide diversity of 0.0224±0.0113. The average pairwise divergences between individuals, within each of the lineages (±SE) were 7.33±3.44, 11.71±5.34, respectively (Table 1).

**Table 1:** Summary of molecular diversity for two lineages.

	n	No. of haplotype	* K*	* H*	* π*	Tajima's *D*		Fu's *F_S_*		Mismatch distribution		
Groups						*D*	*P*	*F_S_*	*P*	*τ*	θ_0_	θ_1_
Pooled	429	402				–	–	–	–	–	–	–
Lineage A	299	274	7.33±3.44	0.999±0.001	0.0138±0.0072	−1.79	0.007	−24.40	0.002	5.50	1.60	99999.00
Lineage B	130	128	11.71±5.34	0.999±0.001	0.0224±0.0113	−0.60	0.323	−24.23	0.000	11.70	0.01	99999.00

Number of individuals (n), number of haplotype, average pairwise differences among individuals (*k*), haplotype diversity (*h*± standard deviation), nucleotide diversity (π± standard deviation) for each grouping of samples, Tajima's *D* and Fu's *F_S_*, corresponding *P*−value, and mismatch distribution parameter estimates for each lineage were also indicated.

**Figure 3:**
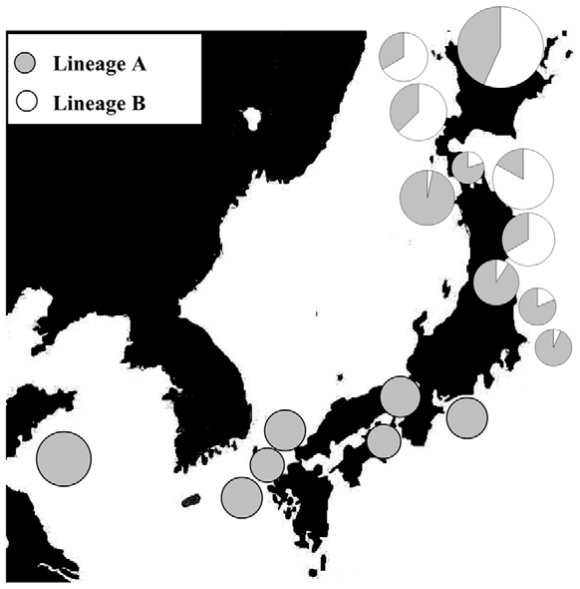
Haplotype frequencies for *A. personatus* populations. The area of circle is proportional to sample size.

**Figure 4:**
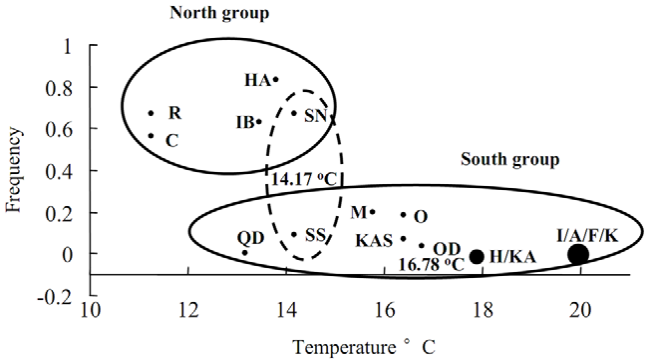
The relationship between the frequency of lineage B and the annual temperature among 17 populations.

There were obviously geographical differences in haplotype frequencies of the two lineages (Fig 3; Fig 4). Lineage A has a more extensive geographical distribution and is present in all 17 populations from Cape Soya to Qingdao. It dominates the south group of *A. personatus* but the frequency declined steadily along the Pacific coast and Sea of Japan coast from south to north. Lineage B was sympatric with lineage A in ten populations, included five populations in south group and all populations in north group, and the frequency of lineage B declined along the coast from Cape Soya to Kashima. The annual sea temperature in lineage A and lineage B varied from 11.26°C to 19.99°C and 11.26°C to 16.41°C, respectively (Table 2). Compared with the haplotype frequency of 17 populations, the relatively stable haplotype frequency was observed in southern populations in south group, including Qingdao population from Yellow Sea (100% for lineage A; absent for lineage B) and populations in north group (17.1%–43.5% for Lineage A, 56.5%–82.9% for lineage B) (Fig 3). The median region between two groups included transition zone of currents in Pacific Ocean (Kashima, Otsuko and Sendai Bay) and Sea of Japan coast off Aomori (Hachinohe and Odose). Two lineages were detected in all four populations in transition zone between Kuroshio Current and Oyashio Current (36°–38°N). The frequency of lineage A significantly declined along the coast from Kashima (93.3%) to Sendai Bay (33.3%) and the lineage B showed the opposite trend. Northward shift of latitude in median region was observed in Sea of Japan. In Sea of Japan coast off Aomori (40°–41°N), the frequency of lineage A declined along the Japan Sea coast from Odose (93.3%) to Ishikari Bay (37.1%), this was similar trend like the transition zone. In both sides of Japanese Island, the median region shows nearly the same annual sea temperature (14.17°C–16.41°C in transition zone; 15.76°C–16.78°C in Sea of Japan). The plot figure based on the frequency of lineage B and annual sea temperature among populations excluding Qingdao showed the obvious separation of the north and south groups (Fig 4). The threshold annual temperature in north group and lineage B were 14.17°C and 16.78°C, respectively.

**Table 2:** Sampling information, number and proportion of individuals and number of haplotypes for the phylogenetic lineages A and B in different populations, annual temperature and ocean current for each population were also showed.

ID	Biogeographic region	Population	Annual temperature	Ocean current	Sample size	Date of collection	Number of individuals in lineage A (proportion, %)	Number of individuals in lineage B (proportion, %)
QD	South group	Qingdao	13.16	/	38	April 2005	38 (100%)	0
A	South group	Ainoshima Island	19.99	Tshushima Current	22	April 2005	22(100%)	0
F	South group	Fukuoka	19.99	Tshushima Current	12	April 2005	12(100%)	0
K	South group	Kanezaki	19.99	Tshushima Current	22	April 2005	22(100%)	0
KA	South group	Kagawa	17.93	Kuroshio Current	11	April 2005	11(100%)	0
H	South group	Hyogo	17.93	Kuroshio Current	24	April 2005	24(100%)	0
I	South group	Ise Bay	19.89	Kuroshio Current	24	May 2005	24(100%)	0
KAS	South group	Kashima	19.06	Transition zone	15	March 2006	14(93.3%)	1(6.7%)
O	South group	Otsuko	16.41	Transition zone	16	May 2004	13(81.3%)	3(18.7%)
SS	South group	Sendai Bay	14.17	Transition zone	22	April 2006	20(90.9%)	2(9.1%)
SN	North group	Sendai Bay	14.17	Transition zone	27	April 2006	9 (33.3%)	18(66.7%)
OD	South group	Oodose	16.78	Tshushima Current	30	May 2006	29(96.7%)	1(3.3%)
M	South group	Mutsu Bay	15.76	Tshushima Current	10	March 2006	8 (80%)	2(20%)
HA	North group	Hachinohe	13.81	Oyashio Current	41	June 2005	7 (17.1%)	34(82.9%)
IB	North group	Ishikari Bay	13.46	Tshushima Current	35	April 2006	13(37.1%)	22(62.9%)
R	North group	Rebun Island	11.26	Tshushima Current	18	June 2006	6(33.3*)	12(66.7%)
C	North group	Cape Soya	11.26	Tshushima Current	62	June 2006	27(43.5%)	35(56.5%)
Total					429		299(69.7%)	130(30.3%)

Network of lineage A was star-like with a dominant haplotype (65.21%) shared by all populations (Fig 2, Table 3). No subclade of lineage A was found in 17 samples. In lineage B, the network represented a “double stars” shape, in that most haplotypes were very closely related to the two common haplotypes, which were distributed in eight populations (Fig 2, Table 4). The two common haplotypes were separated by a single substitution.

**Table 3:** Distribution of transversion haplotypes among populations in lineage A.

Hap	QD	A	F	K	KA	H	I	KAS	O	SS	SN	HA	OD	M	IB	R	C	Total
H3	1																	1
H4	1																	1
H5	27	14	9	16	7	14	14	11	9	12	4	4	18	4	12	5	15	195
H10													1					1
H11	1	2		1			1	1					3					9
H15	2									1				1				4
H16										1								1
H22																	1	1
H23	1	2		1									1				1	6
H24																	1	1
H25							1		2								1	4
H26																	1	1
H27					1												1	2
H28																	1	1
H29						1			1									2
H30						2			1									3
H31	1						1											2
H32	1						1			1								3
H33	2				1	1												4
H34	1												1					2
H35			1	1		1						1	1					5
H36						1							1					2
H37						1												1
H38						1												1
H39						1												1
H40						1												1
H45																1		1
H46			1				1							2				4
H47														1				1
H48		1						1										2
H49								1										1
H56												1						1
H57												1						1
H58													1					1
H59													1					1
Hap	QD	A	F	K	KA	H	I	KAS	O	SS	SN	HA	OD	M	IB	R	C	Total
H60													1					1
H63															1			1
H68											1							1
H69											1							1
H70											2							2
H71											1							1
H72																	1	1
H73																	1	1
H74																	1	1
H75																	1	1
H76																	1	1
H77										1								1
H78										1								1
H79										1								1
H80		1					1			1								3
H81										1								1
H82		1																1
H83		1																1
H84					1													1
H85					1													1
H86							1											1
H87							1											1
H88							1											1
H89							1											1
H9			1															1
H91				1														1
H92				1														1
H93				1														1

**Table 4:** Distribution of transversion haplotypes among populations in lineage B.

Hap	KAS	O	SS	SN	HA	OD	M	IB	R	C	Total	Hap	KAS	O	SS	SN	HA	OD	M	IB	R	C	Total
H1		1	2	4	21					19	47	H42				1					2		3
H2		1									1	H43									1		1
H6				3		1		14	4		22	H44									1		1
H7									1		1	H50					1						1
H8		1		3	1		1			6	12	H51					1						1
H9	1			1	6		1			4	13	H52					1						1
H12								1			1	H53					1						1
H13								1			1	H54					1						1
H14				1						1	2	H55					1						1
H17										1	1	H61				1				1			2
H18										1	1	H62								1			1
H19										1	1	H64				1							1
H20										1	1	H65				1							1
H21										1	1	H66				1							1
H41								4	3		7	H67				1							1

The AMOVA analysis showed that 36.72% of the genetic diversity was found between north and south groups (*P*<0.001). This significant differentiation between groups was consistent with the plot figure based on the frequency of lineage B and annual sea temperature. A very small (0.50%) but significant (*P* = 0.001) amount of genetic diversity was found among populations within groups. The AMOVA analysis also showed that a large and significant genetic differentiation (47.71%, *P*<0.001) was found between Kuroshio Current (including transition zone) and Oyashio Current, a small and non-significant amount of genetic variation (0.15%, *P* = 0.08) was found among populations within the current area. No significant genetic structure was found between the Pacific Ocean and Sea of Japan groups (*F*_CT_ = −0.041, *P* = 0.886), but a high and significant amount of genetic variation was detected among populations within the two sea groups (*F*_SC_ = 0.250, *P*<0.001). Populations within the south group showed no significant structuring (*F*_ST_ = 0.008, *P* = 0.058). Populations within the north group also showed no significant structuring (*F*_ST_ = 0.017, *P = *0.076). Pairwise *F*_ST_ values between 17 populations ranged from −0.020 to 0.513, and most of pairwise *F*_ST_ values between north and south groups were significant after sequential Bonferroni correction except 6 comparisons between groups (Table 5). None of pairwise *F*_ST_ value between populations within south and north groups was significant after sequential Bonferroni correction. Pairwise *F*_ST_ for each lineage was also calculated. For lineage A, none of pairwise *F*_ST_ value between populations was significant after sequential Bonferroni correction. For lineage B, pairwise *F*_ST_ between populations also showed no significant genetic difference after sequential Bonferroni correction.

**Figure 5:**
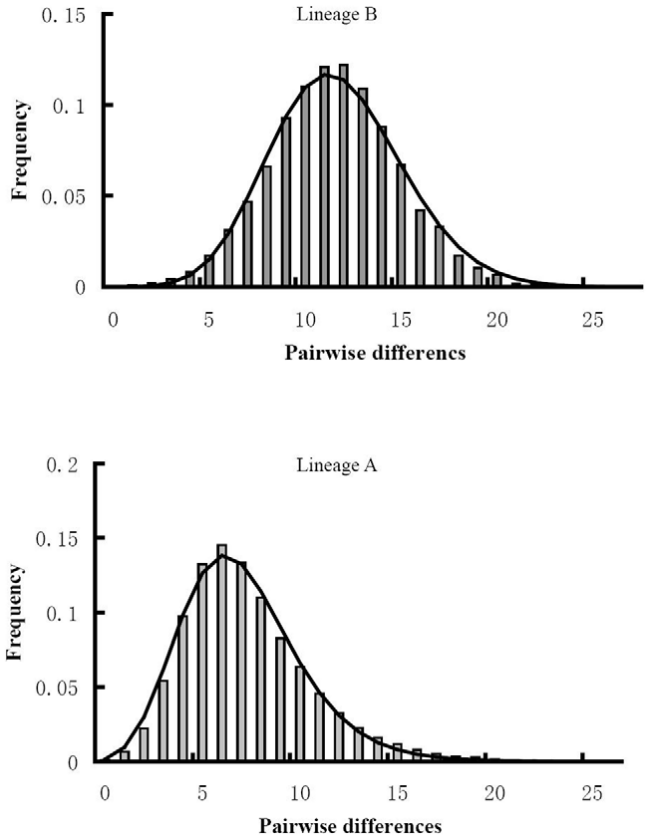
The observed pairwise differences (bars), and the expected mismatch distributions under the sudden expansion model (solid line) for two lineages.

The mismatch distributions for lineage A and lineage B were unimodal, supporting a model of sudden expansion (Fig 5). The *P*_SSD_ and raggedness tests could not reject the expansion hypothesis. For lineage A, it was estimated that population expansion occurred approximately at 129,000 years ago, while for the lineage B this time was 275,000 years ago. The Fu's and Tajima's *D* tests for lineage A and B were negative and significant (Table 1), which indicated population expansion. These were consistent with the star shape networks.

**Table 5:** Pairwise *F*_ST_ (below diagonal) and associated *P* (above diagonal) values among populations of *A. personatus*.

	QD	A	F	K	KA	H	I	KAS	O	SS	SN	OD	M	HA	IB	R	C
QD	–	0.282	0.374	0.549	0.909	0.995	0.788	0.118	0.009	0.103	0.000**	0.507	0.008*	0.000**	0.000**	0.000**	0.000**
A	0.0039	–	0.345	0.473	0.403	0.880	0.844	0.123	0.055	0.520	0.000**	0.545	0.054	0.000**	0.000**	0.000**	0.000**
F	0.0027	0.0051	–	0.463	0.326	0.671	0.129	0.583	0.152	0.266	0.000**	0.847	0.101	0.000**	0.000**	0.000**	0.000**
K	−0.0025	−0.0007	−0.0018	–	0.482	0.955	0.446	0.553	0.071	0.563	0.000**	0.869	0.038*	0.000**	0.000**	0.000**	0.000**
KA	−0.0198	0.0020	0.0068	−0.0020	–	0.973	0.906	0.486	0.118	0.380	0.000**	0.485	0.142	0.000**	0.000**	0.000**	0.000**
H	−0.0167	−0.0125	−0.0083	−0.0177	−0.0295	–	0.947	0.481	0.065	0.604	0.000**	0.922	0.050*	0.000**	0.000**	0.000**	0.000**
I	−0.0071	−0.0122	0.0176	0.0003	−0.0218	−0.0148	–	0.030	0.021*	0.260	0.000**	0.273	0.017*	0.000**	0.000**	0.000**	0.000**
KAS	0.0144	0.0175	−0.0044	−0.0040	−0.0022	−0.0011	0.0284	–	0.115	0.472	0.000**	0.697	0.342	0.000**	0.000**	0.000**	0.000**
O	0.0672	0.0439	0.0421	0.0421	0.0386	0.0354	0.0617	0.0244	–	0.615	0.000**	0.049	0.861	0.000**	0.000**	0.004*	0.006*
SS	0.0123	−0.0017	0.0072	−0.0033	0.0011	−0.0031	0.0074	−0.0029	−0.0122	–	0.000**	0.438	0.606	0.000**	0.000**	0.000**	0.000**
SN	0.4253	0.3826	0.3545	0.3805	0.3523	0.3766	0.4016	0.3031	0.2128	0.2936	–	0.000**	0.020*	0.164	0.459	0.578	0.305
OD	−0.0010	−0.0025	−0.0159	−0.0117	−0.0022	−0.0123	0.0048	−0.0093	0.0391	0.0000	0.3738	–	0.033*	0.000**	0.000**	0.000**	0.000**
M	0.0835	0.0538	0.0463	0.0588	0.0409	0.0501	0.0809	0.0045	−0.0451	−0.0163	0.1602	0.0502	–	0.000**	0.022*	0.044*	0.057
HA	0.5305	0.4962	0.4769	0.4948	0.4777	0.4920	0.5131	0.4311	0.3447	0.4167	0.0115	0.4872	0.2968	–	0.021*	0.075	0.004*
IB	0.3726	0.3356	0.3105	0.3314	0.3184	0.3318	0.3551	0.2636	0.1736	0.2499	−0.0070	0.3253	0.1325	0.0477	–	0.721	0.452
R	0.4112	0.3684	0.3447	0.3631	0.3398	0.3604	0.3921	0.2786	0.1783	0.2613	−0.0129	0.3579	0.1264	0.0323	−0.0181	–	0.742
C	0.2939	0.2641	0.2410	0.2604	0.2467	0.2595	0.2803	0.2079	0.1197	0.1912	0.0012	0.2530	0.0823	0.0640	−0.0037	−0.0153	–

* , significant at *P*<0.05 by the permutation test; **, significant *P* values after Bonferroni correction.

## Discussion

Sharp phylogeographical breaks in all species indicate limits to dispersal over both contemporary and historical time scales [25]. The present results revealed significant genetic divergences in *A. personatus* among samples along the Japanese Island with the haplotype clusters on the NJ tree and the reduced median-network. Many authors suggest that Pleistocene glaciations were the most significant event to shape the phylogeographic mitochondrial DNA patterns and population structure in marine fish species [8], [9]. Two distinct lineages were found in *A. personatus*, and the origin of two lineages is probably related to the Pleistocene coastal glaciations. Lineage A and B dominated the south group and north group, respectively, indicating origin in Pacific Ocean and Sea of Japan. Like Pacific herring and Pacific cod, *A. personatus* originated in subpolar region and colonized southward to Japanese Island [26]. The high nucleotide diversity in north group supported the subpolar region origination. With the advance of glaciers, the Sea of Japan was almost isolated from the Pacific Ocean, and a physical barrier to gene flow was created between the Sea of Japan and Pacific Ocean. Considering the earlier glacial terminations (Term II, Term III, Term IV, and Term V) approximately 130, 240, 325, and 420 kyr ago [27], the provisional molecular clock yields that the divergence of two lineages (453,000 years) occurred in glacial period. The isolation of the Sea of Japan and Pacific Ocean might create the two lineages of *A. personatus*. During the postglacial period, two lineages began to secondary contact. The secondary contact of two lineages was supported by isozyme data. Isozymed data have showed that the frequency of α-*Gpdh^F^* is about 0.3 in north group, about zero in south group and about 0.08 in transition zone [22], [28]. This frequency of α-*Gpdh^F^* among populations showed the similar trend like the frequency of lineage B. The polymorhism of α-*Gpdh* in north group and transition zone might be nuclear imprint of secondary contact. Previous studies have demonstrated that the Sea of Japan was isolated from the Pacific Ocean and other marginal seas during glaciations, and was a refugium for marine species, such as Japanese sea bass *Lateolabrax japonicus*, redlip mullet *Chelon haematocheilus* [8], [10] and marine invertebrates [29], [30]. Our findings also revealed that historical factors were the most significant event to shape phylogeography in Northwestern Pacific.

Significant haplotype frequency changes of two lineages along the Japan coast showed high limited gene flow between south and north groups. The AMOVA and pairwise *F*_ST_ showed significant genetic structures between the groups, supporting the hypothesis of genetic differentiation between Kuroshio and Oyashio Currents, but contracting the hypothesis of strong gene flow among locations in the Sea of Japan coast off Japanese Island. The distribution of haplotypes may result from both current and historical process. Thus, an insight into population structure is also necessary for the proper inference on the contemporary factors in shaping population genetic structure. Circulation patterns, temperature regimes, coastal topography, environmental requirements and life histories of species are suggested as important factors to affect the dispersal of species in marine environments [31]–[33]. The candidate barrier prevented the dispersal between the south and north group in *A. personatus* is the different sea temperature derived by the pattern of oceanic circulation in Northwestern Pacific. In Pacific side of Japan, lineage B was restricted in the transition zone and the Oyashio Current area. In the Sea of Japan with no cold current, northward shift of latitude was observed in lineage B. The same annual sea temperature was observed in southern populations of lineage B in both sides of Japan. The distribution of north group showed similar trend like the lineage B. Northward shift of latitude was also observed in north group in the Sea of Japan. Compared with results, it is obvious that the sea temperature is a key factor to prevent the dispersal of lineage B between groups, despite the ocean currents caused shift of latitude in secondary region compared with both sides of Japanese Island.

The differences in seawater temperature, strong correlation of these differences with distribution of lineage and groups suggest an adaptation to local thermal regimes. Santos et al. (2006) states that water temperature is one of the main factors that affect the distribution of marine fish (reviewed in [34]). Genetic studies on the king weakfish, *Macrodon ancylodon*, a member of the family Sciaenidae found along the Atlantic coast of South America, showed that there are two deep divergent lineages representing tropical and subtropical groups. The separation of the tropical and subtropical clades suggests a pattern of allopatric differentiation, attributed to local thermal adaptation [34]. Similar pattern are also observed in *Fundulus heteroclitus* [7]. *F. heteroclitus* are found along the East Coast of North America, with populations distributed along a steep thermal gradient. Studies of genetic variation in this species suggest that there are two distinct genotypes (Northern and Southern), with a broad zone of admixture between the two genotypes in intermediate latitudes. The genetic variation between northern and southern of *F. heteroclitus* was regarded as a means of adapting to a changing environment. This genetic pattern of *F. heteroclitus* was similar to *A. personatus*. *A. personatus* is a cold-water species and is sensitive to sea temperature. It starts aestivation, when the water temperature exceeds the tolerance limit of *A. personatus*. The duration of aestivation declined from south to north along the Japan coast. For example, aestivation duration in Sendai Bay (August–November) is shorter than that in Ise Bay (May-November) (Hashimoto, 1991). No aestivation was observed in Cape Soya [17]. The habit of aestivation in south group was thought to acquire after glacial period as an adaptation to local rising water temperature [16]. The lineage B with the northern origin might be more sensitive to sea temperature than lineage A. So the distribution of lineage B was restricted by high sea temperature (the annual sea temperature of 16.78°C might be the threshold temperature in lineage B) and spread with the cold water current (Oyashio Current). The distribution of north group was also restricted by high sea temperature (the annual sea temperature of 14.21°C might be the threshold temperature). However, we cannot confirm reproductive isolation between north group and south group, despite limited gene flow were detected in two samples cohabiting in Sendai Bay. Due to the high sea temperature barrier in the southern part of Japan, the lineage B is absent and only lineage A was detected in Qingdao population, which belongs to south group, despite the Yellow Sea has a suitable temperature for lineage B.

However, the genetic differentiation among populations within two groups and two lineages were non-significant, despite a very small and significant amount of genetic diversity was found among populations within groups. Most of *F*_ST_ values between populations within the North or South group are not statistically significant. Only considering the lieage A or lineage B individuals, there are no significant values of *F*_ST_ among pairs of populations. This indicated that the gene flow between populations might be high without sea temperature barrier. Considering the lack of long migration in species, the ocean current within groups might be responsible for this homogeneity in groups.

The isolation of Sea of Japan and Pacific Ocean resulted in two lineages of *A. personatus*. During the glacial periods, the populations of *A. personatus* isolated in Pacific Ocean (Lineage A) migrated south along the costal waters off the Japan Islands with the low sea temperature and weak Kuroshio Current, and finally entered the southern coast of Japanese Island; the *A. personatus* populations isolated in Sea of Japan (Lineage B) might benefit the low sea temperature and the Sea of Japan had been colonized by this lineage B. After the termination of glaciation, the communication between Sea of Japan and Pacific Ocean was created with the rising sea level. The lineage A obtained the aestivation to adapt to the rising sea temperature in southern Japanese Island. The lineage A also expanded its distribution northward to second contact with the lineage B with the strengthen Kuroshio Current in Pacific Ocean and Tsushima Current in the Sea of Japan [11]. This lineage also entered the Yellow Sea. The rising sea temperature and the Tsushima Current flow through Tsushima trait into the Sea of Japan with warm water reduced distribution of lineage B in the Sea of Japan and canceled the chance of lineage B to enter the Yellow Sea through the opened Tsushima strait. However, the lineage B expanded its southern range in Pacific Ocean with strong Oyashio Current flow with cold water. These currents and sea temperature shaped the present secondary contact zone in the Sea of Japan and Pacific Ocean. The calculated expansion time indicated the expansion of lineage A occurred in the termination of Term II glaciation, and the expansion of lineage B occurred in the Term III glacial period. The expansion time is consistent with our hypothesis. However, the status of north group and south group was not confirmed by our results. Nuclear markers such as AFLP or SSR are needed to resolve the status of the two groups.

Our results illuminate the combination of historical process and contemporary factors to shape present population structure in *A. personatus*. The process of secondary contact between two lineages and the distribution of north group and south group were heavily influenced by sea temperature. The ocean current reinforces and weakens the sea temperature barrier. This gives us a lesson about the cold-water species how respond with the ocean current and sea temperature. This also provides information about the mechanism of ocean current on population structure in marine species. Our results suggested that annual sea temperature could serve as an indicator for the group identification. The fishery management of *A. personatus* can benefit from our result.

## Materials and Methods

All experimental procedures involving fish were approved by the Institutional Animal Care and Use Committee of Zhejiang Ocean University under 2012-01.

No specific permits were required in sampling. No specific permissions were required for these locations/activities in this study. The sampling location is not privately-owned or protected in any way. This study did not involve endangered or protected species.

### Sampling and sequencing

**Figure 6:**
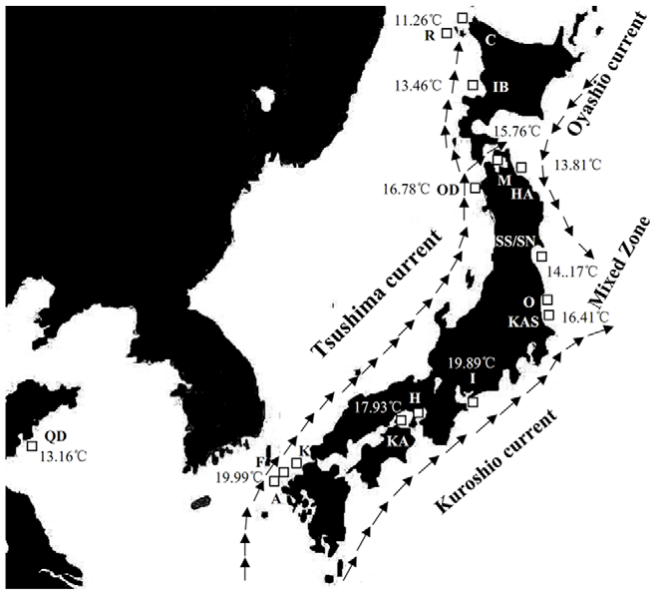
The study area depicting sample locations, schematic map of currents and the annual sea temperature.

The sample size consists of 429 individuals from 17 samples across its natural distribution, from the southern extreme of its range off the coast of Kyushu and its northern limit Cape Soya. Sampling information and sampling locations are listed in Table 2 and Fig 6, respectively. The south group and north group were identified by the descriptions of Okamoto et al. [23], [28] and Hashimoto and Kawasaki [22]. Two samples (SS, SN), which belong to north and south groups, were collected in the same location in Sendai Bay. Oceanographic features including currents, annual sea surface temperatures (Because of no long migration in adults and vertical migration in night, the annual surface temperature was chosen.) at the sampled localities are also indicated in Fig 6 and Table 2. The annual sea temperature was available in JOC (http: www.jodc.go.jp). Muscle was obtained and preserved in 95% ethanol or frozen for DNA extraction. Genomic DNA was isolated from muscle tissue by proteinase K digestion followed by a standard phenol–chloroform method. DNA was subsequently resuspended in 100 μL of TE buffer. PCR primers specific to *A. personatus* were designed from the universal primers to amplify the first hypervariable segment of the mtDNA control region [35]. The primer sequences are DL-S, 5′-CCC ACC ACT AAC TCC CAA AGC-3′ (forward) and DL-R, 5′-CTG GAA AGA ACG CCC GGC ATG-3′ (reverse). PCR was carried out in 50 μL volumes containing 1.25 U *Taq* DNA polymerase (Takara Co., China), 20 ng template DNA, 200 nmol/L forward and reverse primers, 200 μmolL/L of each dNTPs, 10 mmol/L Tris, pH 8.3, 50 mmol/L KCl, 1.5 mmol/L MgCl_2_. The PCR amplification was carried out in a Biometra thermal cycler under the following conditions: 3 min initial denaturation at 94°C, and 40 cycles of 45 s at 94°C for denaturation, 45 s at 50°C for annealing, and 45 s at 72°C for extension, and a final extension at 72°C for 10 min. All sets of PCR included a negative control reaction tube in which all reagents were included, except template DNA. PCR product was purified with Gel Extraction Mini Kit (Watson BioTechnologies Inc., Shanghai). The purified product was used as the template DNA for cycle sequencing reactions performed using BigDye Terminator Cycle Sequencing Kit (ver. 2.0, PE Biosystems, Foster City, California), and sequencing was conducted on an ABI Prism 3730 (Applied Biosystems) automatic sequencer with both forward and reverse primers. The primers used for sequencing were the same as those for PCR amplification.

### Data analysis

Sequences were edited and aligned using Dnastar software (DNASTAR Inc., Madison, USA). Molecular diversity indices such as number of haplotypes, polymorphic sites, transitions, transversions, and indels were obtained using the program ARLEQUIN (ver. 2.000) [36]. Haplotype diversity (*h*), nucleotide diversity (π) and the mean number of pairwise differences (*k*), and their corresponding variances were calculated following Nei (1987) as implemented in ARLEQUIN [37]. Nucleotide sequence evolution models were evaluated using likelihood-ratio tests implemented by Modeltest v.3.06 [38].

Genetic distances were generated for phylogenetic reconstruction with MEGA4.0 using the model of Tamura and Nei [39] given by the Modeltest. Among site rate heterogeneity was corrected with the shape parameter of gamma distribution (Γ = 0.677). The neighbor-joining tree of the haplotype was constructed using MEGA and evaluated with 1000 bootstrap replicates. Haplotypes of *A. personatus* used in the phylogenetic analysis were representatives of transversion haplotypes. In addition, genealogical relationships were examined based on reduced datasets that contained only the transversions, by constructing haplotype networks using median-network approach [40].

Genetic differentiation between pairs of population samples was evaluated by pairwise fixation index (*F*_ST_). The significance of the *F*_ST_ was tested by 10,000 permutations in ARLEQUIN. To further examine hierarchical population structure as well as the geographical pattern of population subdivision, we used analysis of molecular variance (AMOVA). We conducted AMOVA analysis with two groups representing the north group and south group. The division of groups is shown in Table 2. Two additional AMOVA were conducted to test geographical variation. First, test the role of the Kuroshio Current, and Oyashio Current with two groups representing the two currents; second, test whether *A. personatus* was portioned into two groups, representing the Sea of Japan and Pacific Ocean. When multiple comparisons were performed, *P* values were adjusted using the sequential Bonferroni procedure [41]. A plot figure was made to infer the relationship between the frequency of lineage B and the annual temperature.

The historical demographic expansions were examined by two different approaches. First the *D* test of Tajima and *F*_S_ test of Fu were used to test if the neutrality holds [42], [43]. Significant negative *D* and *F*_S_ statistics can be interpreted as signatures of population expansion. Historic demographic expansions were investigated by examination of frequency distributions of pairwise differences between sequences (mismatch distribution), which is based on three parameters: *θ*_0_, *θ*_1_ (*θ* before and after the population growth) and τ (time since expansion expressed in units of mutational time) [44]. The distribution is usually multimodal in samples drawn from populations at demographic equilibrium, but it is usually unimodal in populations following a recent population demographic expansion and population range expansion. The concordance of the observed with the expected distribution under the sudden expansion model of Rogers was tested by means of a least squares approach [44]. Both mismatch analysis and neutrality tests were performed in ARLEQUIN. For distribution that did not differ significantly (*P*>0.05) from the expectation of the sudden expansion model, the parameter of the demographic expansion was τ estimated by a generalized nonlinear least square approach, and confidence intervals was computed using a parametric bootstrap approach. The values of τ were transformed to estimates of real time since expansion with the equation τ* = *2*ut*, where *u* is the mutation rate for the whole sequence under study and *t* is the time measured in years since expansion.

The molecular clock for the control region seems to vary among major taxonomic groups of marine fishes (reviewed in [9]). *A. personatus* with short generation time, high metabolic rate and small body size like Japanese anchovy might have a rapid molecular clock. In the present study, sequence divergence rate of 8%/Myr was applied for the control region sequences of *A. personatus*.
